# Hepatic Pin1 Expression, Particularly in Nuclei, Is Increased in NASH Patients in Accordance with Evidence of the Role of Pin1 in Lipid Accumulation Shown in Hepatoma Cell Lines

**DOI:** 10.3390/ijms24108847

**Published:** 2023-05-16

**Authors:** Machi Kanna, Yusuke Nakatsu, Takeshi Yamamotoya, Akifumi Kushiyama, Midori Fujishiro, Hideyuki Sakoda, Hiraku Ono, Koji Arihiro, Tomoichiro Asano

**Affiliations:** 1 Department of Medical Science, Graduate School of Medicine, Hiroshima University, Hiroshima 734-8551, Japan; 2 Department of Pharmacotherapy, Meiji Pharmaceutical University, 2-522-1, Kiyose 204-8588, Japan; 3 Division of Diabetes and Metabolic Diseases, Nihon University School of Medicine, Tokyo 173-8610, Japan; 4 Division of Neurology, Respirology, Endocrinology and Metabolism, Department of Internal Medicine, Faculty of Medicine, University of Miyazaki, Miyazaki 889-1692, Japan; 5 Department of Clinical Cell Biology, Graduate School of Medicine, Chiba University, Chiba 260-8670, Japan; 6 Department of Anatomical Pathology, Hiroshima University Hospital, Hiroshima 734-8551, Japan

**Keywords:** Pin1, NASH, liver biopsy

## Abstract

Our previous studies using rodent models have suggested an essential role for Pin1 in the pathogenesis of non-alcoholic steatohepatitis (NASH). In addition, interestingly, serum Pin1 elevation has been reported in NASH patients. However, no studies have as yet examined the Pin1 expression level in human NASH livers. To clarify this issue, we investigated the expression level and subcellular distribution of Pin1 in liver specimens obtained using needle-biopsy samples from patients with NASH and healthy liver donors. Immunostaining using anti-Pin1 antibody revealed the Pin1 expression level to be significantly higher, particularly in nuclei, in the livers of NASH patients than those of healthy donors. In the samples from patients with NASH, the amount of nuclear Pin1 was revealed to be negatively related to serum alanine aminotransferase (ALT), while tendencies to be associated with other serum parameters such as aspartate aminotransferase (AST) and platelet number were noted but did not reach statistical significance. Such unclear results and the lack of a significant relationship might well be attributable to our small number of NASH liver samples (*n* = 8). Moreover, in vitro, it was shown that addition of free fatty acids to medium induced lipid accumulation in human hepatoma HepG2 and Huh7 cells, accompanied with marked increases in nuclear Peptidyl-prolyl cis-trans isomerase NIMA-interacting 1 (Pin1), in accordance with the aforementioned observations in human NASH livers. In contrast, suppression of *Pin1* gene expression using siRNAs attenuated the free fatty acid-induced lipid accumulation in Huh7 cells. Taken together, these observations strongly suggest that increased expression of Pin1, particularly in hepatic nuclei, contributes to the pathogenesis of NASH with lipid accumulation.

## 1. Introduction

The number of patients with non-alcoholic steatohepatitis (NASH) are rising in most countries. Patients with NASH account for approximately 3–5% of the global population [1]. NASH, which is closely related to obesity and diabetes, and accumulation of fat and inflammation coexist in the liver [1]. It is a matter of serious concern that NASH promotes hepatic fibrosis, hepatic cirrhosis, and, eventually, liver malignancies. Generally, NASH is diagnosed based on liver biopsy, and its severity is evaluated based on the degree of steatosis, inflammation, ballooning of hepatocytes, and fibrosis [2]. One of the major diagnostic standards is the Matteoni classification [3]. The stages of this classification are as follows: 1. steatosis, 2. steatosis and invasion of inflammatory cells, 3. steatosis and ballooning of hepatocytes, and 4. steatosis and ballooning with mallory denk bodies or fibrogenesis.

The “multiple hit” theory was proposed as a mechanism underlying NASH development. Firstly, insulin resistance results from lipid peroxidation [4,5]. After receiving the first hit, hepatocytes are vulnerable to second-hit damage, including oxidative stress [4] and the induction of hepatocyte injury and inflammation. Recently, this second hit came to be viewed as a “parallel multiple hit” [5] process which involves the triggering of multiple steps in the disease process. Despite progress in clinical research, no satisfactorily effective therapies have yet been developed.

Our previous study demonstrated that Pin1 is markedly increased in the livers of NASH model mice fed either a high-fat diet or a methionine choline-deficient diet. Importantly, Pin1 KO mice are highly resistant to the development of NASH or non-alcoholic fatty liver disease (NAFLD) [6]. Pin1 is one of the isomerase enzymes and regulates various signal transductions through isomerization of target proteins at the peptide bond between their phosphoserine/threonine- and proline-containing motifs [7]. Pin1 contributes not only to lipid accumulation, but also inflammation and fibrosis [6,8]. Therefore, it is likely that Pin1 plays an essential role in the development of NASH based on experiments using mice, and, indeed, an elevated serum Pin1 concentration was demonstrated in NASH patients. However, to our knowledge, published information regarding the Pin1 expression level in human NASH livers is as yet lacking. 

This study aimed to clarify the expression level and subcellular distribution of Pin1 in NASH livers, as compared with normal livers. In addition, we used hepatoma HepG2 and Huh7 cell lines to investigate how stimulation with free fatty acids affects Pin1, as well as how suppression of Pin1 using siRNA alters free fatty acid-induced lipid accumulation. 

## 2. Results

### 2.1. NASH Patients

Liver biopsy samples were obtained from eight patients with NASH and four healthy donors for liver transplantation. The average ages of the patients with NASH and the healthy subjects were 67.1 and 41.7 years, respectively. Serum values of AST, ALT and γ-glutamyl transpeptidase (γ-GTP) were significantly higher in the NASH patients than the healthy subjects, while platelet and serum albumin concentrations did not differ significantly between the two groups (Table 1).

### 2.2. Immunohistochemistry Using Pin1 in NASH Biopsy Samples

**Figure 1:**
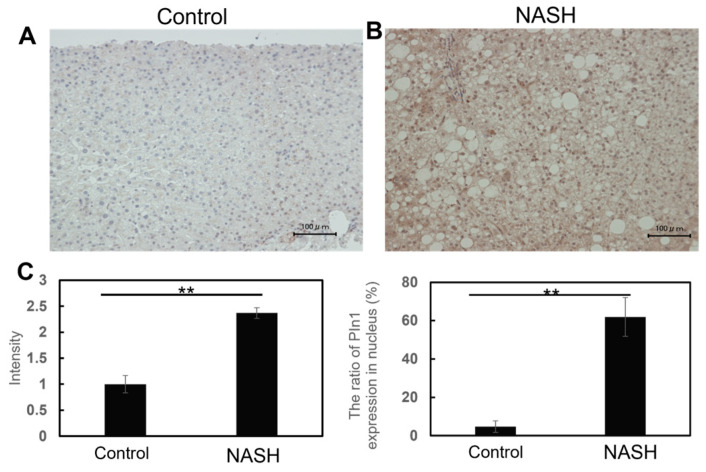
Pin1 expression was markedly elevated in NASH livers. Immunohistochemical staining of liver biopsy samples using anti-Pin1 antibody was performed for eight patients with NASH and four healthy liver donors. Nuclei were stained with hematoxylin. Representative samples from one NASH and one normal liver are shown (**A**,**B**), while staining data for all samples are presented in Supplementary Figure S1. Based on immunohistochemical staining with anti-Pin1 antibody, nuclear Pin 1 expression levels were analyzed and compared with the eight NASH and four normal liver samples, as shown in (**C**). Data are presented as means SEM. **; *p* < 0.01.

Expression levels and subcellular localizations of Pin1 in the liver specimens were analyzed based on the immunochemical staining of biopsy samples. All biopsy samples were stained with mouse IgG as a control, and the absence of significant staining with control IgG was confirmed (Supplementary Figure S1). Staining of the samples of healthy donor subjects with anti-Pin1 antibody showed almost undetectable or minimal signals (Figure 1A,B, and lower four panels of Supplementary Figure S2). In contrast, Pin1 was abundant in both the nuclei and cytosol of all NASH samples (Figure 1B and upper eight panels of Supplementary Figure S2). Regarding the subcellular localization of Pin1, in NASH livers, Pin1 was more abundant in the nuclei than the cytosol. The staining intensity with anti-Pin1 antibody was analyzed, and the results were presented as a graph (left panel of Figure 1C). In addition, the percentages of Pin1-strongly positive nuclei were compared with the liver specimens from healthy controls and NASH patients (right panel of Figure 1C). These results clearly showed markedly elevated nuclear Pin1 in the NASH livers compared to normal livers.

### 2.3. Relationships of the Nuclear Pin1 Level with Various Blood Parameters in NASH

**Figure 2:**
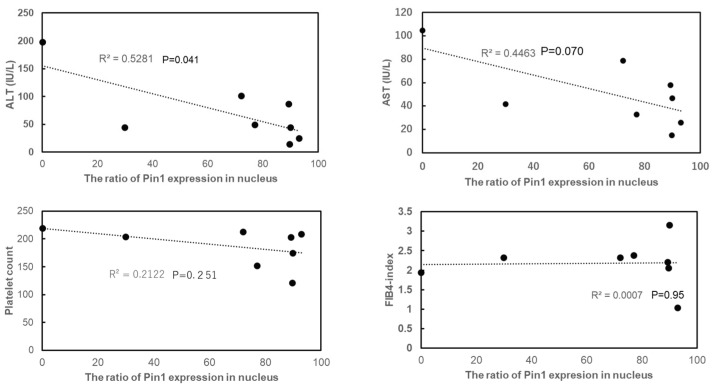
Relationships of nuclear Pin1 level with various serum parameters. Relationships between nuclear Pin1 level and ALT, AST platelet number and FIB-4 index were analyzed based on 8 NASH samples.

Subsequently, in the NASH samples, we analyzed the relationships between the nuclear Pin1 amount and various serum parameters related to liver functions. ALT values were negatively related to the amount of nuclear Pin1. In addition, a relatively weak tendency, which did not reach statistical significance, for negative relationships between AST and platelet number with the nuclear Pin1 amount was noted. No significant relationship was detected between the fibrosis-4 (FIB-4) index and nuclear Pin1 amount (Figure 2). However, we are not highly confident in these results due to the relatively small number of samples.

### 2.4. Lipid Droplet Formation Was Induced via Palmitic Acid and Oleic Acid in HepG2 and Huh7 Cells

**Figure 3:**
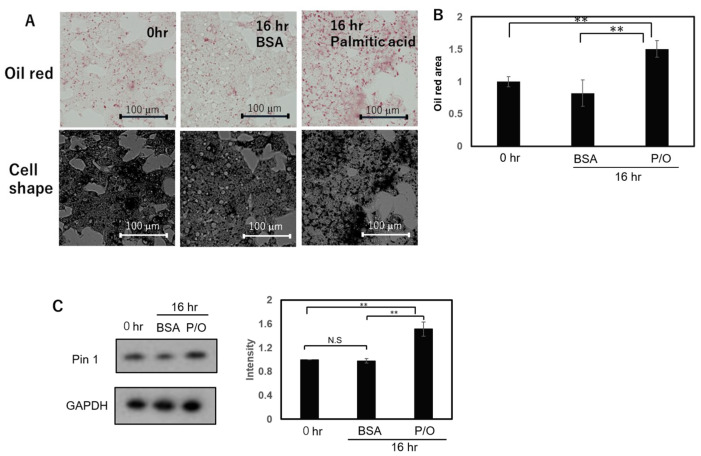
Effects of free fatty acid stimulation on lipid accumulation and Pin expression level in HcpG2 cells. HepG2 cells were treated with 50 µM palmitic acid and 25 µM oleic acid for 16 h. Lipid accumulation was then examined via oil-red O staining (**A**), and stained area was calculated for each of groups prior to treatment with BSA alone or free fatty acid plus BSA (**B**). In HepG2 cells, Pin1 expression was examined via immunoblotting using anti-Pin1 antibody and quantified via Image J (**C**). Each experiment was caried out three times (*n* = 4), and data are presented as means ± SEM. **: *p* < 0.01. N.S: not significant.

**Figure 4:**
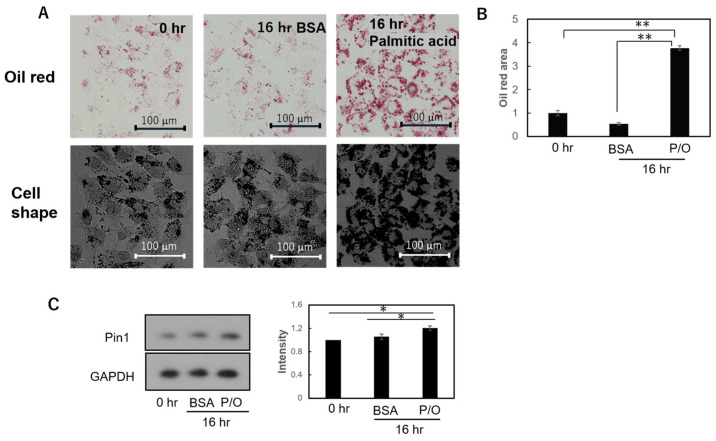
Effects of free fatty acid stimulation on lipid accumulation and Pin1 expression level in Huh7 cells. Huh7 cells were treated with 50 µM palmitic acid and 25 M oleic acid for 16 h. Lipid accumulation was then examined via oil-red O staining (**A**), and stained area was calculated for each of groups prior to treatment with BSA alone or free fatty acid plus BSA (**B**). In Huh7 cells, Pin1 expression was examined via immunoblotting using anti-Pin1 antibody and quantified via Image J (**C**). Each experiment was carried out three times (*n* = 4), and data are presented as means ± SEM. *; *p* < 0.05, **; *p* < 0.01.

We examined whether or not Pin1 is upregulated, as in NASH biopsy samples, in hepatoma cells with lipid accumulation. The addition of free fatty acids, including 50 µM palmitic acid and 25 µM oleic acid, for 16 h induced an increase in lipid droplets in hepatoma HepG2 and Huh7 cells, based on the oil red O staining results (Figure 3A and Figure 4A). These oil droplets were quantified via Image J ver. 1.53k and are shown in Figure 3B and Figure 4B, respectively.

In addition, Pin1 expression was shown, through immunoblotting with anti-Pin1 antibody, to be significantly increased (Figure 3C and Figure 4C). These in vitro observations in hepatoma-cultured cells are in good agreement with the results obtained from human NASH biopsy samples (Figure 1). We also investigated whether Pin1 overexpression affects lipid accumulation. However, there was no difference in lipid accumulation between overexpression and normal expressions (Supplementary Figure S3).

### 2.5. Treatment with Palmitic and Oleic Acids Increased Nuclear Pin1 in Huh7 Cells

**Figure 5:**
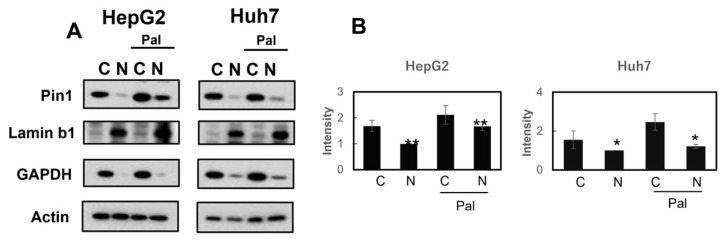
Subcellular localizations of Pin1 in Huh7 and HepG2 cells with or without free fatty acid stimulation. Cells were treated with 50 µM palmitic acid and 25 µM oleic acid for 16 h. Lamin bland GAPDH were used as markers in nucleus and cytosol, respectively (**A**). Protein expressions were quantified via Image J (**B**). *; *p* < 0.05, **; *p* < 0.01.

The nuclear and cytosolic fractions of Huh7 and HepG2 cells, with or without free fatty acid stimulation for 16 h, were prepared and subjected to immunoblotting using anti-Pin1 antibody. Anti-laminin and anti-NADPH antibodies were used as the nuclear and cytoplasmic markers, respectively. Nuclear Pin1 was shown to be significantly increased in response to free fatty acid stimulation, as compared with the control (Figure 5A,B).

**Figure 6:**
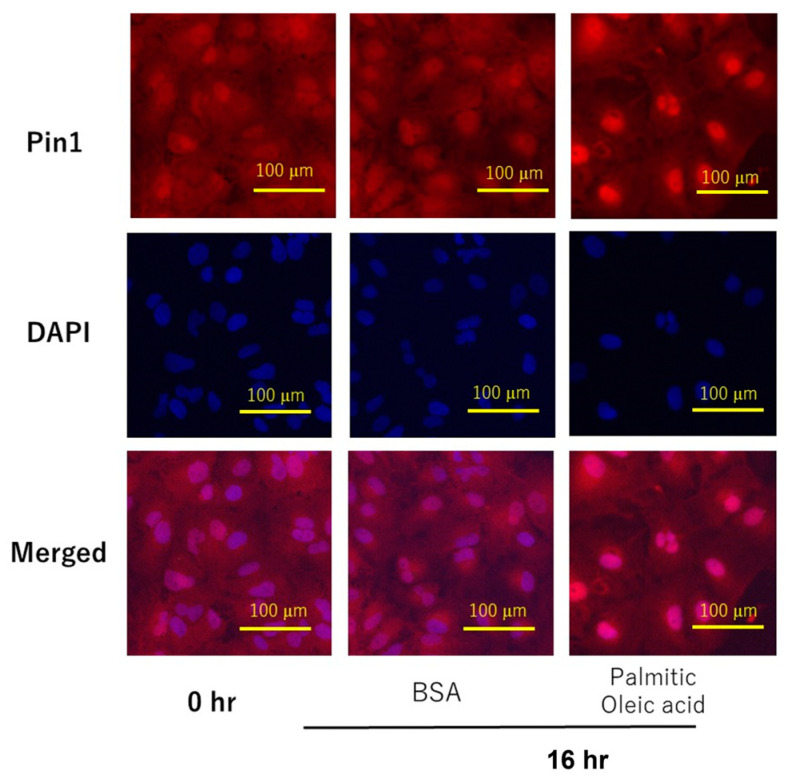
Subcellular localizations of Pin1 in Huh7 cells with or without free fatty acid stimulation. Huh7 cells were treated with 50 µM palmitic acid and 25 µM oleic acid for 16 h. Immunohistochemical staining using anti-Pin1 antibody and DAPI staining were performed.

Subsequently, immunofluorescent staining with anti-Pin1 antibody was carried out in Huh7 cells with or without free fatty acid stimulation for 16 h, while nuclei were co-stained with DAPI (Figure 6). Nuclear Pin1 was markedly increased in response to free fatty acid stimulation, as compared with the control. On the other hand, in HepG2 cells, Pin1 was located mainly in the nuclei, even in the basal state, and, thus, no clear effect of free fatty acid stimulation was detectable via immunofluorescent staining.

### 2.6. Pin1 siRNA Suppressed Lipid Droplet Production in Response to Free Fatty Acid Stimulation

**Figure 7:**
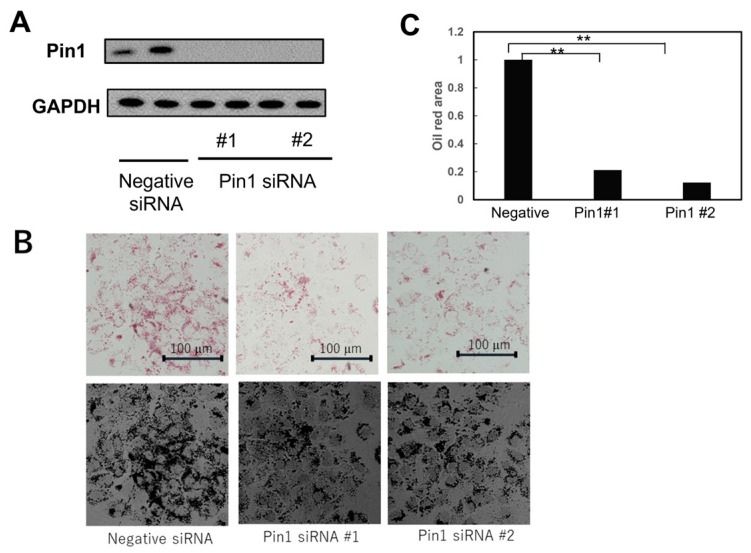
Pin1 suppression using its siRNAs reduced free fatty acid-induced lipid accumulation inHuh7 cells. Huh7 cells were treated with either two Pini siRNAs or control siRNA, and incubated in medium containing 50 µM palmitic acid and 25 µM oleic acid for 16 h. Pin1 expression was reduced using the Pin1 siRNAs, as demonstrated via immunoblotting using anti-Pin1 antibody (**A**). Huh7 cells treated with either two Pin siRNAs or control siRNA were subjected to oil-red 0 staining (**B**), and the lipid droplet arca was quantified via Image J (**C**). Experiments were carried out three times (*n* = 4), and data are presented as means ± SEM. **; *p* < 0.01.

To investigate the contribution of Pin1 to free fatty acid-induced lipid accumulation, Huh7 cells were treated with Pin1 siRNA, which suppressed Pin1 expression (Figure 7A). Accumulation of lipid droplets, as detected via oil-red-O staining, was markedly reduced through treatment with Pin1 siRNAs (Figure 7B). Lipid droplets were quantitatively analyzed via ImageJ and are shown in Figure 7C. These results clearly support the essential role of Pin1 in lipid accumulation.

## 3. Discussion

Pin1 is a unique enzyme which enhances isomerization of the peptide bond between phosphorylated serine/threonine and proline in target proteins [9,10]. Pin1 i not only physiologically beneficial but also causative pathogenic roles in several disorders via modulating the functions of its numerous target proteins. For example, Pin1 KO mice reportedly develop Alzheimer disease because Pin1 binds to and promotes the degradation of Tau protein [11,12,13]. In contrast, increased Pin1 expression was reported to be related to the aggressiveness of malignancies and poor patient outcomes [14,15,16]. This tumorigenesis modulated via Pin1 occurs in conjunction with JNK [17].

Previously, we reported that Pin1 expression was markedly increased in the liver, muscle, and fat tissues of obese or high-fat diet-fed mice [18]. Importantly, Pin1 KO mice were shown to be highly resistant to the development of obesity and NASH [6].

Therefore, we considered it very likely that Pin1 is overexpressed in human NASH livers, while elevated serum Pin1 concentrations were reported in patients with NASH [19]. However, definitive information regarding the Pin1 expression level in human NASH livers is as yet lacking. In this study, we analyzed eight samples from NASH livers and four samples from healthy livers, and it was obvious that the Pin1 expression level was higher and showed particularly marked nuclear localization in the livers of NASH patients compared to those of healthy subjects.

Next, we focused on the amount of nuclear Pin1 in NASH liver samples as it was difficult to measure the amount of cytosolic Pin1. Pin1 reportedly contains nuclear localization signal, the core domain of which is between amino acids 61 and 70 [20]. However, Pin1 is actually distributed in both the nucleus and the cytoplasm, which is mainly affected through being phosphorylated. Pin1 phosphorylation of Ser16 through PKA inhibits the binding ability of Pin1 to MPM-2, thereby suppressing Pin1 nuclear localization [21]. WW domain phosphorylation plays a critical role in regulating phosphoserine binding activity and Pin1 function [21]. In contrast, Pin1 phosphorylation at Ser138 via MLK3 increases Pin1 nuclear localization [22]. Mixed-lineage kinase 3 phosphorylates prolyl-isomerase Pin1, thereby regulating its nuclear translocation and cellular functions [22]. Therefore, the subcellular distribution of Pin1 is regulated via post-translational modifications.

The relationships between nuclear Pin1 and most of the serum parameters were not statistically significant due to the small number of NASH samples (*n* = 8), although ALT values showed a negative relationship with the amount of nuclear Pin1. In addition, nuclear Pin1 tended to show negative relationships with AST and platelet number. No significant relationship was detected between the FIB-4 index and nuclear Pin1. Had a much larger number of NASH samples been available for the analysis, a significant relationship might have emerged. Further study is warranted to assess the potential relationship between nuclear Pin1 and the stage of NASH. Nevertheless, it was clearly demonstrated that Pin1 is dramatically overexpressed in NASH, as compared with normal livers.

Subsequently, we performed in vitro experiments using hepatoma cell lines. It was demonstrated that free fatty acid-induced lipid accumulation accompanied an increase in Pin1, particularly nuclear Pin1, and that suppression of Pin1 expression using its siRNAs significantly reduced free fatty acid-induced lipid accumulation in both HepG2 and Huh7 cells. In contrast, lipid accumulation with this fatty acid did not increase significantly in response to overexpression of Pin1. Huh7 and HepG2 are cancer cells, and their Pin1 expression levels are, thus, markedly elevated compared to those in normal hepatocytes. For this reason, in our view, Pin1 overexpression failed to increase lipid accumulation, despite siRNA-mediated suppression of Pin1 markedly reducing it. Taken together, these observations highlight the importance of Pin1 in NASH development with lipid accumulation, which was confirmed through our experiments using both human clinical samples and cultured hepatoma cell lines.

Prior studies revealed multiple mechanisms plausibly explaining how Pin1 is involved in the lipid accumulation observed in cells. Firstly, Pin1 binds to IRS-1 and Akt, and thereby enhances insulin signaling, leading to lipogenesis [18]. On the other hand, Pin1 binds to and suppresses phosphorylation, which is a necessary step in enzymatic activation, of AMP-activated protein kinase, which is an important regulator of lipolysis [23]. Furthermore, Pin1 also acts on the enzymes regulating lipid metabolism. Stated concretely, Pin1 enhances the activity of the key enzyme fatty acid synthetase on one hand, while also promoting the degradation of adipose triglyceride lipase, which is a lipolytic enzyme [24], on the other hand. Pin1 also regulates SREBP1-C, which is a key factor in lipogenesis [25,26]. Reflecting the numerous aforementioned actions of Pin1, Pin1 KO mice showed the phenotype of being highly resistant to the development of obesity or NASH [6]. However, the mechanism through which overnutrition and/or high-fat diet feeding induces Pin1 overexpression remains unclear, and is an important question awaiting resolution.

In summary, this study is the first study to clearly show that Pin1 expression is increased in human NASH liver specimens. We also confirmed that Pin1 plays a critical role in the pathogenesis of NASH development.

## 4. Materials and Methods

### 4.1. Cell lines and Medium

Two hepatocellular carcinoma cell lines—HepG2 and Huh7 cells—were cultured in the Dulbecco’s Modified Eagle Medium (DMEM; Nissui pharmaceutical, Tokyo, Japan) supplemented with 10% fetal calf serum, as well as 0.6 g/L glutamine, 0.1% NaHCO_3_, 0.1 mg/mL streptomycin, and 0.06 mg/mL penicillin in 5% CO_2_, at 37 °C.

### 4.2. Fatty Acid Treatment

Palmitic acid or oleic acid was dissolved at a concentration of 100 mM in 100% ethanol. A total of 850 μL of 0.14 g/mL bovine serum albumin (BSA; FUJIFILM Wako pure chemical corporation) in 1 M Tris-HCl (pH 8.0) was then added to 150 μL of fatty acid solution (Palmitic acid 100 μL, Oleic acid 50 μL), and mixed via vortexing. These mixtures were filtrated via a 0.45 μm filter.

Cells were cultured for 24 h in a 24-well plate. Fatty acids were then added to a cell plate. The final concentrations of fatty acids were 50 μM palmitic acid and 25 μM oleic acid. Cellular lipid accumulation was visualized via Oil Red O staining [27].

### 4.3. Immunohistochemistry Staining

Liver biopsy samples were obtained from 8 patients with NASH and 4 healthy liver donors, who received treatment in Hiroshima University Hospital. These samples were sectioned and slides were prepared for microscopic observation. The slides were washed 3 times each with xylene and ethanol for 3 min and then with 95% ethanol. After paraffin removal, we washed the samples with water for 3 min. Antigen activation was then performed through soaking the slides in 10 mM citric acid (pH 6.0) for 20 min at 97 °C. Samples were washed in phosphate buffered saline (PBS) 3 times. To achieve endogenous peroxidase removal, the samples were soaked in 0.3% H_2_O_2_ dissolved in MeOH for 15 min. After the samples were washed with PBS, they were reacted with 2 μg/μL of Pin1 antibody (Santa Cruz biotechnology, Dallas, TX, USA) in Can get sol B (Toyobo, Osaka, Japan) at room temperature. As a control, the same amount of mouse IgG was used. After 1 h of incubation, the samples were washed with PBS 3 times. Biotin-labelled secondary antibody was then added to the samples, followed by incubation for 30 min, and, finally, reaction with an avidin–biotin-labelled enzyme complex (VECTASTAIN ABC Kit; Vector Laboratories, Newark, CA, USA) for the subsequent 30 min. The samples were washed with PBS at each step. As a substrate for horseradish peroxidase (HRP), we used ImmPACT™ DAB Peroxidase (Vector Laboratories, Newark, CA, USA). After staining with hematoxylin, the samples were soaked in water for 5 min. To remove the water, we placed the samples in ethanol 3 times and then twice in xylene. This project was approved by the ethics committee of Hiroshima University, and all individual participants provided fully informed written consent.

### 4.4. Separation of Nucleus and Cytosol

Cells were cultured overnight in 6-well plates and then suspended in PBS and scraped using a scraper.

Cell suspensions were centrifuged at 500× *g* for 3 min. For separation of cytosol and nucleus, we used NE-Per Nuclear and cytoplasmic extraction reagent (Thermo Scientific, Tokyo, Japan). Precipitates were resuspended using cytoplasmic extraction reagent 1. Suspensions were mixed via vortexing and stored on ice for 10 min. Cytoplasmic extraction reagent 2 was added into suspensions. Suspensions were mixed via vortexing. Cells were centrifuged at 15,000× *g* for 5 min. Supernatants, which included cytosolic protein, were transferred to new tubes and stored at −80 °C. The precipitates were resuspended in nuclear extraction reagent. This suspension was mixed via vortexing every 10 min and stored on ice for 40 min. The suspensions were centrifuged at 15,000× *g* for 5 min. The nuclear fraction supernatant was immediately removed and stored at −80 °C.

### 4.5. Western Blotting

The cell lysates were subjected to SDS-PAGE in 15% polyacrylamide gel and then transferred onto a PVDF membrane (Nihon Millipore K.K., Tokyo, Japan). The membranes were incubated in 3% skim milk for 1 h. The membranes were then washed 3 times with PBS and incubated with the anti-Pin1, anti-GAPDH (Proitentech, Tokyo, Japan) and laminb1(Proteintech, Tokyo, Japan) antibody overnight. After the antigen-antibody reactions, the membranes were washed 3 times with PBS. The HRP-conjugated secondary antibodies were then added, and the reactions were demonstrated via enhanced chemiluminescence solution (GE Healthcare, Tokyo, Japan).

### 4.6. Fluorescent Staining

HepG2 or Huh7 cells were cultured in 35 mm dishes. After fatty acid treatment for 16 or 24 h, medium was removed via an aspirator, and the cells were fixed through adding 10% formaldehyde to the dishes. The cells were then washed 3 times with PBS. After washing the cells, PBS with 0.1% triton was added to the dishes. The cells were again washed with PBS. Blocking was performed for 1 h using 3% goat serum. Pin1 antibody in Can get sol B was applied overnight. The cells were then washed 3 times with PBS. Alexa546 (Thermo Fischer Scientific, Tokyo, Japan) in 3% goat serum, used for 1h, served as the secondary antibody. DAPI was used for nuclear staining.

### 4.7. Suppression of Pin1 Expression via siRNA

For the preparation of a complex of Opti-MEM and siRNA, 100 μL of Opti-MEM and 1.2 pmol siRNA (#1 “CAGTATTTATTGTTCCCACAA”, #2 “CTCGTCCTGGCGGCAG-GAGAA” were added to 24-well or 3.5-cm glass bottom dishes. Lipofectamine and 1 μL of RNAiMax (Invitrogen, Waltham, MA, USA) were then added on the same plate, followed by incubation for 15 min at room temperature. Finally, 500 μL of 1 × 105 cells were added to the complex.

### 4.8. Overexpression of Pin1 in HepG2 and Huh7

A strain overexpressing Pin1 was constructed via transforming PcDNA3.1-Stag-Pin1. Cells were transfected with PcDNA 3.1 as a control. For preparation of the complex, 50 μL of Opti-MEM, 1 µg/µL plasmid and x-tream gene HP DNA transfection reagent (Merck, Tokyo, Japan) were mixed, followed by incubation at room temperature for 15 min. Finally, this complex was added to the cell preparations.

### 4.9. Statistical Analysis

Results were expressed as means ± S.E. Statistical significance was assessed using the *t*-test. *p* < 0.05 was taken to indicate a statistically significant difference.

## 5. Conclusions

We investigated the expression level and subcellular distribution of Pin1 in liver specimens obtained using needle-biopsy samples from both patients with NASH and healthy liver donors. The level of Pin1 expression was found to be significantly elevated, particularly in nuclei. Since Pin1 was specifically expressed in NASH patients, Pin1 has potential for the clinical diagnosis of NASH.

Moreover, in human hepatoma HepG2 and Huh7 cells, Pin1 increased in nuclei after treatment with fatty acids, in accordance with the above observations in human NASH livers. In contrast, suppression of Pin1 gene expression using siRNAs attenuated the free fatty acid-induced lipid accumulation in Huh7 cells. Taken together, these observations strongly suggest that increased Pin1 expression, particularly in hepatic nuclei, contributes to the pathogenesis of NASH, which is characterized by lipid accumulation.

## Figures and Tables

**Table 1:** Clinical data in NASH and control patients.

	NASH	Control
Age	66.1 ± 3.80	41.7 ± 8.30
AST	47 ± 9.75	19.3 ± 0.98 **
ALT	70.3 ± 19.6	9.67 ± 1.18 **
γ-GTP	72.8 ± 11.14	1.96 ± 24.28 **
PLT	183 ± 11.88	196 ± 24.28
ALB	4.07 ± 0.045	4.53 ± 0.22

**; *p* < 0.01.

## Data Availability

The datasets generated and analyzed during the current study are available from the corresponding author on reasonable request.

## Supplementary Materials

The following supporting information can be downloaded at: https://www.mdpi.com/article/10.3390/ijms24108847/s1.
