# Supplementary material for: Hepatic Pin1 Expression, Particularly in Nuclei, Is Increased in NASH Patients in Accordance with Evidence of the Role of Pin1 in Lipid Accumulation Shown in Hepatoma Cell Lines
Source: Int J Mol Sci. 2023 May 16;24(10):8847. doi: 10.3390/ijms24108847 (PMC10218692; doi:10.3390/ijms24108847)
Supplement: Supplementary file 1 [file ijms-24-08847-s001.zip › ijms-2337664-supplementary.pdf]

Supplementary figure S1

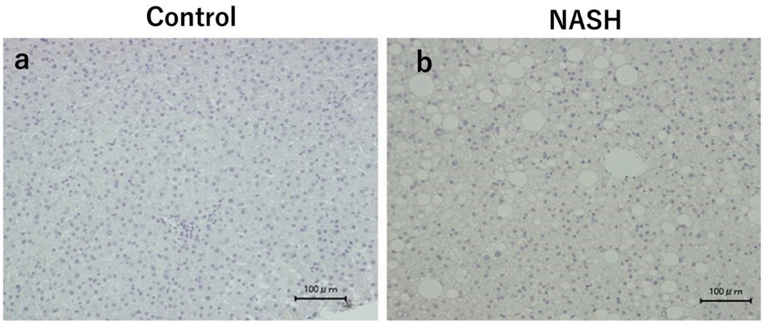

SupFig.Sl Mouse IgG antibody was used as a control. Each panel corresponds to those in Fig.1. Images show the effects of intrinsic biotin to have been excluded.

Supplementary figure S2

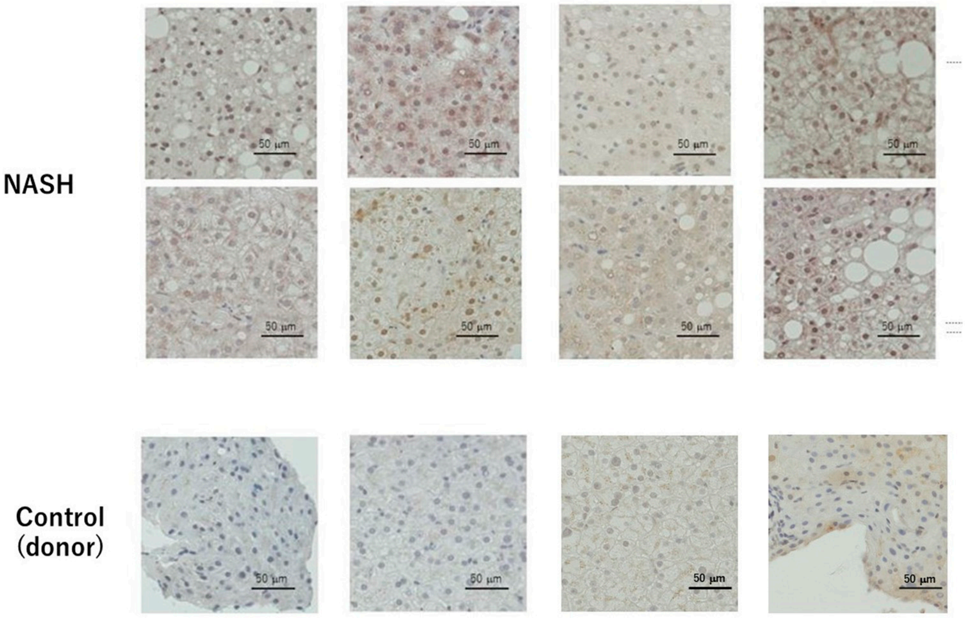

Supplementally figure S2
Pin1 expressions in liver biopsy samples from NASH patients and donors.
Pin1 was expressed strongly in the nucleus and cytosol of NASH samples.
Donor samples showed weak expression only in the cytosol.

Supplementary figure S3

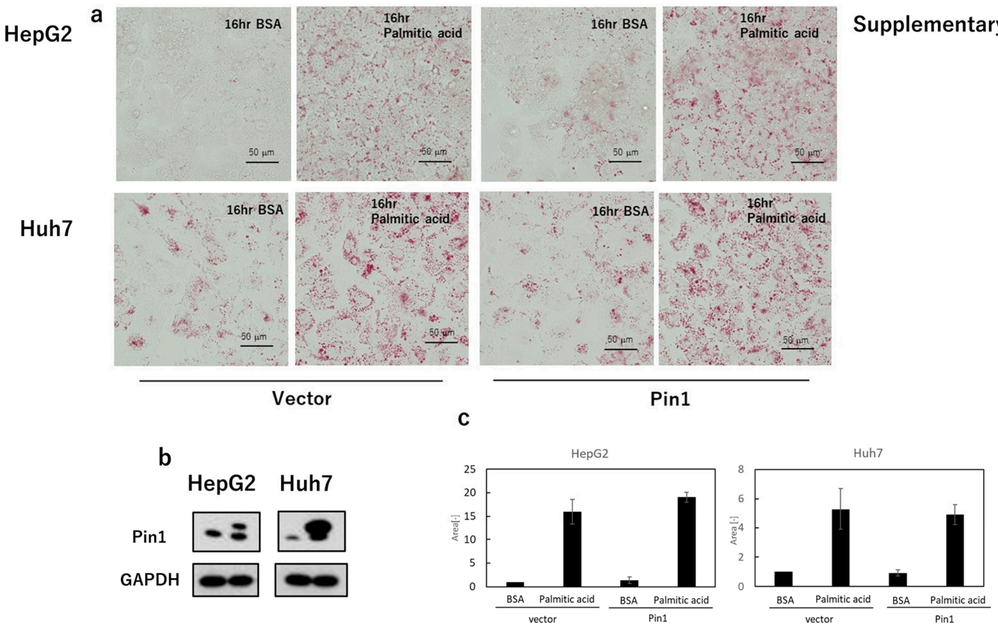

Sup Fig. S3 Effects of free fatty acid stimulation on lipid accumulation in cells overexpressing Pin1

Cells were treated with 50 μM palmitic acid and 25 μM oleic acid for 16 hrs. Then, lipid accumulation was examined by oil-red O staining (A) and the stained area was calculated as overexpression of Pin1 and vector
